# Offloading Strategies Used for Plantar Diabetic Foot Ulcers and Their Outcomes in Real-Life Clinical Practice

**DOI:** 10.3390/jcm14113834

**Published:** 2025-05-29

**Authors:** Afram Rumanes, Jaap J. van Netten, Kor H. Hutting, Lisette J. E. W. C. van Gemert-Pijnen, Jeff G. van Baal

**Affiliations:** 1 Department of Surgery, Hospital Group Twente, 7609 PP Almelo, The Netherlands; 2 Department of Persuasive Health Technology, University of Twente, 7522 NB Enschede, The Netherlands; 3 Department of Rehabilitation, Amsterdam UMC, University of Amsterdam, Meibergdreef 9, 1105 AZ Amsterdam, The Netherlands; 4 Amsterdam Movement Sciences, Program Rehabilitation and Development, Amsterdam UMC, University of Amsterdam, 1105 AZ Amsterdam, The Netherlands; 5 School of Public Health, Queensland University of Technology, Brisbane, QLD 4059, Australia; 6 ZGT Academy, Hospital Group Twente, 7600 SZ Almelo, The Netherlands; 7 Scharenborg Groep, 10, 7555 SK Hengelo, The Netherlands

**Keywords:** diabetes mellitus, foot ulcers, offloading, ulcer healing

## Abstract

**Introduction:** International guidelines describe offloading to facilitate healing as a cornerstone in the treatment of diabetes-related foot ulcers. In present-day clinics, various offloading devices are used. The aim of this paper is to describe the effectiveness in healing of different offloading devices used in real-life clinical practice in patients with diabetes-related foot ulcers. **Methods:** A retrospective cohort study of 235 patients with a plantar foot ulcer in one diabetic foot centre of expertise was used. Clinical outcomes were determined during a follow-up period of 12 months. Groups were defined according to the types of offloading. Univariate and multivariate analysis was performed to assess ulcer-related outcomes in different offloading devices. **Results:** Of the 235 patients, 3% were treated with a Total Contact Cast (TCC), 9% with an ankle-high removable device, 32% with a custom-made orthopaedic shoe, 16% with a bandage shoe, and 39% with felted foam. Patients who received a bandage shoe or felted foam had a higher UT classification (Stage D in 21% and 18%, respectively, *p* = 0.001) and more ulcers per foot (13% and 5%, respectively, *p* = 0.002). The overall healing rate at 12 weeks was 33% and was not significantly different between the offloading device groups (*p* = 0.255). Healing rates at 20 and 52 weeks were 51.5% and 77%. **Conclusions:** Removable ankle-high offloading devices, orthopaedic shoes, bandage shoes, and felted foam are the most frequently used for plantar diabetic foot ulcers in clinical practice. This seems to be the result of various physician- and patient-related factors such as logistical reasons, patient factors, and severity of complicated ulcers. Diabetic foot ulcer healing after 12 weeks, 20 weeks, and 1-year follow-up were consistent with previous observational studies.

## 1. Introduction

Diabetic foot ulcers are a threatening and common complication of diabetes mellitus [1]. Studies show a lifetime incidence between 19% and 34% of diabetic foot ulcers, as well as a recurrence rate of more than 75% within 5 years [2]. Diabetic foot ulcers significantly increase the risk of infection and amputation, and reduce patients’ quality of life and mobility. In patients with diabetes, the risk of death is twice as high in patients with foot ulcers compared to those without [3,4]. Around 10% of patients die within 30 days of a major amputation, and more than 70% of the patients with diabetes-related amputations will die within 5 years [3,4,5]. Economic consequences are major ($58 billion annual in the United States and $770 million in the United Kingdom) [6,7], not only because of the treatment of the immediate ulcer episode, but also because of the treatment of subsequent episodes, as well as the social effect [8]. It is therefore important to heal a diabetic foot ulcer. Diabetic foot ulcers are mainly caused by elevated levels of mechanical pressure acting on the foot, mostly due to foot deformity in combination with a lack of foot sensation due to peripheral neuropathy [9,10,11]. Almost 50% of diabetic foot ulcers are located at the plantar surface of the foot.

International guidelines prescribe offloading the pressure as an imperative aspect and cornerstone in the treatment of plantar diabetic foot ulcers [12]. For this goal, various offloading methods are available [13,14]. It is recommended in international guidelines to use non-removable knee-high offloading devices for plantar forefoot or midfoot diabetic foot ulcers that are not complicated by infection or ischemia, or by mild infection or mild ischemia only [12]. The recommendation for a non-removable knee-high offloading device is based on superiority in meta-analyses of RCTs comparing these devices with removable (knee-high or ankle-high) alternatives [15], as well as evidence from a large Australian cohort showing shorter healing times in patients treated with non-removable knee-high offloading devices [16].

Other offloading methods that are also used in daily practice and are described in international guidelines include removable knee-high or ankle-high devices (including ankle-high custom-made footwear), alternative devices (e.g., bandage shoes), or even just felted foam [15]. The most recent systematic review and meta-analyses on this topic provide evidence for knee-high removable and ankle-high removable offloading being equal to each other regarding outcome, with little-to-no difference in proportions of ulcers healed (RR 1.00, 95% CI 0.86–1.16) [12,15]. In this meta-analysis, ankle-high custom-made orthopaedic shoes were also considered as an ankle-high removable offloading device [15]. Consequently, these devices are seen as the second option in treating plantar foot ulcers, when knee-high non-removable devices are contraindicated.

However, as also stated by the most recent guidelines of the International Working Group on Diabetic Foot (IWGDF), offloading RCTs have focused almost exclusively on the treatment of non-complicated neuropathic plantar forefoot ulcers [12]. Few data are available on the value of offloading in healing plantar foot ulcers complicated by infection or ischaemia, in rearfoot ulcers, or in non-plantar ulcers [12]. Yet these ulcers together are now arguably more common than ‘purely’ neuropathic plantar forefoot and midfoot ulcers [12]. More insight in offloading outcomes from a mixture of foot ulcers representative of daily clinical practice is needed.

From previous observations in real-life clinical practice, it is known that the recommended treatment of a non-removable knee-high offloading device is rarely (2–23%) used [17,18,19,20,21]. However, these observational studies with data on offloading devices used in real-life clinical practice do not provide healing rates of the offloading devices used in their mixture of uncomplicated and complicated diabetic foot ulcers [15]. The RCTs that are conducted regarding non-removable knee-high offloading devices show 12-week healing rates around 80–90% [11,22,23,24]. Contrary, the aforementioned Australian observational cohort study found a 12-week healing rate of 41.5% in the total population. The effect of knee-high offloading had an odds ratio of 1.34, suggesting healing percentages (much) lower than 80% in real-life clinical practice [16]. No clinical outcomes of offloading devices are available from other observational studies. As a result, there is limited knowledge on the healing outcomes following treatment with different offloading devices in real-life clinical practice.

There is still a gap in the knowledge about the different offloading methods used in daily practice and the effectiveness of these devices in ulcer healing. The aim of this study is to describe the offloading devices used in daily clinical practice in a specialized diabetic foot centre and the effectiveness of the different offloading devices in patients with a plantar diabetic foot ulcer.

## 2. Materials and Methods

### 2.1. Study Design

A retrospective cohort study was conducted in Hospital Group Twente, a regional centre of expertise for diabetic foot care in Almelo, The Netherlands. The STROBE guidelines were used for reporting, providing a framework for transparent and complete reporting of the design, conduct, and results of an observational study [25]. A minimal follow-up of 12 months had to be available for inclusion in this study, or until a patient died within the follow-up period. All study activities were performed according to the Declaration of Helsinki and the Dutch Personal Data Protection Act. This study was not assessed by a medical ethical committee because it does not fall within the scope of the Dutch Medical Research Involving Human Subjects Act.

### 2.2. Study Population

All patients (>18 years) with diabetes mellitus, presenting with one or more plantar foot ulcers in the outpatient clinic of the diabetic foot centre in Hospital Group Twente, were included between December 2014 and May 2020. We selected the patient cohort that was used in previous studies [26,27]. For the current study, we included all referred patients with a plantar ulcer, including infected/ischemic ulcers. We excluded patients if they were already included with another ulcer during the study period, and patients with dorsal ulcer(s) only. A plantar diabetic foot ulcer was defined as ‘a break of the skin of the foot that involves as a minimum the epidermis and part of the dermis on the underside or weight-bearing surface of the foot’ [28]. Patients with a deep or infected and/or ischemic ulcers (i.e., Texas classification A3, B, C, and D) are considered complicated ulcers in our cohort. Uncomplicated ulcers are considered ulcers that are not infected/ischemic, and are superficial (i.e., Texas A1 and A2). A separate analysis was performed for the uncomplicated ulcers and for the complicated ulcers.

Diabetic foot ulcers were diagnosed by members of the multidisciplinary diabetic foot team in Hospital Group Twente, which has more than 20 years of experience in treatment of diabetic foot ulcers. The members of the multidisciplinary team consisted of vascular surgeons, podiatrists, wound care nurses, casting technicians, specialists in internal medicine, specialists in rehabilitation medicine, radiologists, and orthopaedic shoe technicians.

### 2.3. Treatment of Included Patients

Patients received standard diabetic foot ulcers treatment [1,29,30]. Treatment included, among others, the treatment of the infection with antibiotics, the treatment of peripheral artery disease (PAD) if indicated, regular wound debridement and wound dressings, and education. All patients received a form of offloading as treatment for a plantar diabetic foot ulcer. The following five categories of offloading devices were used and are shown in Appendix A.

#### 2.3.1. Knee-High Non-Removable Offloading Devices

Most used device in this category was the Total Contact Cast (TCC) [31].

#### 2.3.2. Knee-High Removable Offloading Devices

Most used device in this category was the Bi-valved TCC (BTCC). This is a TCC that is made removable by sawing it anterolaterally and anteromedially with a plaster saw into two valves, as described in detail elsewhere [31]. To connect the two U-shaped parts of the BTCC, a cohesive bandage was used.

#### 2.3.3. Ankle-High Removable Offloading Devices

Devices used as a removable ankle-high device included the MABAL shoe, forefoot offloading shoe (FOS), and custom-made orthopaedic shoes with felted foam, allowing ankle mobility. The MABAL shoe is a fibreglass cast shoe, applied by a skilled technician, which can be rendered to facilitate walking by combining it with a roller walking sole [32]. The FOS is a prefabricated shoe, with a specific shape with a wedge design and the outsole portion missing in the forefoot, especially designed for relieving areas of the forefoot [33]. Custom-made orthopaedic shoes are prescribed by a specialist in rehabilitation medicine, and custom-made for an individual patient by a certified orthopaedic shoe technician. Both are part of the multidisciplinary diabetic foot ulcer team in Hospital Group Twente [34].

#### 2.3.4. Other

In addition to the abovementioned offloading devices, ‘other offloading treatments’ may be chosen. These included felted foam in combination with a bandage shoe or felted foam in patients’ own regular shoe. Felted foam can be used to redistribute and relieve pressure away from one part of the foot, when shaped and applied appropriately [22]. The felted foam was replaced twice a week.

Medical personnel took different patient and ulcer factors into account in the decision-making to determine the offloading device, such as patient preference, time-related factors at the outpatient clinic, patient adherence, and ulcer-related factors such as location of the ulcer and infected/ischaemic ulcers; these were, however, not specifically captured in this study. In consultation with the patient, a specific offloading device was chosen. Experienced casting technicians of the diabetic foot unit were available to apply offloading devices, with casting methods described in detail elsewhere [31,33].

### 2.4. Data Collection

Data regarding relevant comorbidities, diabetic foot ulcers, treatment, and clinical outcomes were collected by the investigator. Diabetic foot ulcer location was expressed using the following plantar regions: hallux, 2nd digit, 3rd digit, 4th digit, 5th digit, forefoot, midfoot. The University of Texas (UT) system was used to classify diabetic foot ulcers [35]. Loss of protective sensation (peripheral neuropathy) was investigated using a 10 g monofilament and the loss was defined as absence of pressure sensation [30]. End-stage renal disease was defined as a glomerular filtration rate < 30 mL/min/1.73 m^2^ or renal replacement therapy [36]. Cardiovascular disease was defined as a history of percutaneous coronary intervention of one or more of the coronary arteries or a coronary artery bypass graft and a myocardial infarction. Cerebrovascular disease was defined as a history of either an ischemic cerebrovascular accident or a cerebral haemorrhage. Peripheral arterial disease (PAD) was defined as an Ankle-Brachial-Index (ABI) ≤ 0.9 [37].

### 2.5. Outcomes

Primary outcomes were the healing rate at 12 weeks, 20 weeks, and 1 year. Secondary outcomes were time to ulcer healing, ulcer recurrence, and ulcer-free survival days. Diabetic foot ulcer healing was defined as an intact skin for a minimum of 2 weeks (with or without prior amputation). Diabetic foot ulcer recurrence was defined as development of a new diabetic foot ulcer within the 1-year follow-up. Ulcer-free survival days were considered to be all the days a patient was alive and ulcer-free (i.e., all ulcers healed) during the follow-up period [38].

### 2.6. Statistical Analyses

Descriptive statistics were used to analyse the baseline patients’ disease- and ulcer-related characteristics, and ulcer outcomes. Continuous variables were presented as mean (SD) in case of normal distribution of the data, or median [IQR] in case of non-normal distribution. Categorical data were presented as a number (percentage). Differences in baseline characteristics between groups were tested using Chi-square tests, Student t-tests or Mann–Whitney U tests, depending on the characteristics of the variables. Univariate and multivariate cox regression analyses with backward elimination were performed to identify independent predictors. Statistical significance was set at a *p*-value < 0.05. Variables with a *p* value < 0.15 in the univariate analysis were considered potential predictors and were entered into the multivariate analysis. A multivariate logistic regression analysis was performed with ulcer healing as dependent variable, and type of offloading device as independent variable, correcting for potential confounders. Kaplan–Meier curves were used to visualise the healing time for diabetic foot ulcers stratified per type of offloading. Statistical analysis was performed using SPSS Statistics for Windows, version 24.0 (IBM Corp, Armonk, NY, USA).

## 3. Results

### 3.1. Baseline Characteristics

In this cohort, 235 patients were included. Baseline characteristics are shown in Table 1 and Table 2. No signs of ischaemia and infection and a superficial ulcer (UT A1) was present in 34% (n = 80) of the cohort. Ulcers complicated by ischaemia and/or infection were present in 55% (n = 130). The hallux was the most common location of the ulcer (43%, n = 100). The majority of the patients were treated with felted foam (39%, n = 92) or an orthopaedic shoe (32%, n = 75). Patients with ulcers on the rearfoot were treated predominantly with felted foam. A total 3% (n = 8) of the patients were treated with a TCC. Patients treated with felted foam, a bandage shoe, or with an orthopaedic shoe had a significantly higher age when compared to patients treated by TCC or ankle-high removable offloading (*p* < 0.001). Furthermore, patients with PAD were predominantly treated by patients with a bandage shoe or felted foam compared to 0% of the patients treated with TCC (*p* = 0.003). Patients with poorly controlled diabetes (higher HbA1c) were more often treated with TCC or ankle-high removable offloading (*p* = 0.006), and more patients had type 1 diabetes (*p* = 0.003) in these offloading groups compared to the other offloading groups. Although not always statistically significant, patients treated with TCC or an ankle-high removable device had hardly any cerebrovascular disease, little cardiovascular disease, and no ESRD compared to the other offloading groups. Treatment with a bandage shoe and felted foam as the offloading device was more common in people with more complex wounds (patients with multiple ulcers, UT stage C and D, or UT grade 3; Table 2).

### 3.2. Treatment Outcome—All Patients

Treatment outcomes of all included patients are shown in Table 3. Diabetic foot ulcer healing after 12 weeks follow-up was 33% (n = 78); no statistically significant differences were found between the offloading device groups (*p* = 0.255). Diabetic foot ulcer healing after 20 weeks follow-up was 52% (n = 121), and at that timepoint, patients treated with felted foam (55%, n = 51) or an orthopaedic shoe (59%, n = 55) had a higher healing rate compared to the other offloading groups (*p* = 0.013). Diabetic foot ulcer healing after 1-year follow-up was 77%, and at that timepoint, patients treated with orthopaedic shoes (84%) or with a TCC (88%) had a higher healing rate compared to the other groups (*p* = 0.003).

The baseline variables that were univariately associated with ulcer healing and type of offloading device with a *p* < 0.15 were included in the multivariate analysis model. After correction for confounders, the following variables remained significant in correlation with ulcer healing at 20 weeks: cardiovascular disease (*p* = 0.016; odds ratio [OR] = 0.438; 95% confidence interval [CI] = 0.224–0.857), number of ulcers (*p* = 0.020; OR = 0.593; 95%CI = 0.381–0.922), wound stage (*p* = 0.004; OR = 0.641; 95%CI = 0.474–0.867), and wound grade (*p* = 0.015; OR = 0.596 95%CI = 0.392–0.905).

After correction for confounders, the following variables remained significant at 1 year: cerebrovascular disease (*p* = 0.033; OR = 0.436; 95%CI = 0.203–0.936), cardiovascular disease (*p* = 0.032; OR = 0.494; 95%CI = 0.259–0.940), number of ulcers (*p* = 0.041; OR = 0.690; 95%CI = 0.483–0.985), and wound grade (*p* = 0.041; OR = 0.669; 95%CI = 0.454–0.984). The results of the univariate and multivariate analysis are included in Appendix B

**Figure 1:**
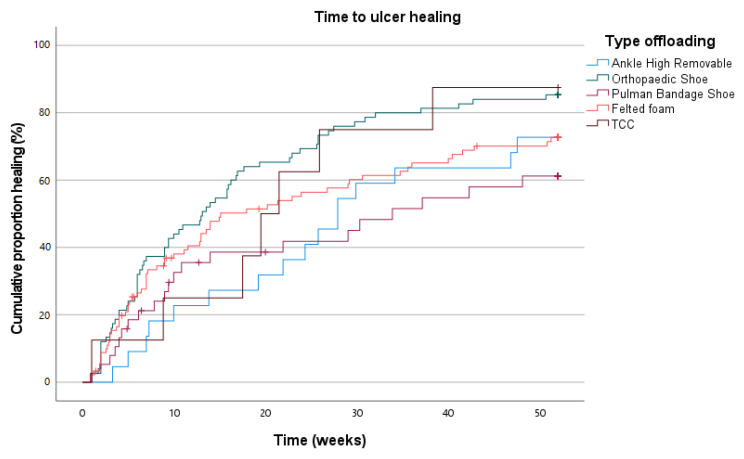
Time to ulcer healing in all patients.

A Kaplan–Meier curve depicting time to healing is shown in Figure 1. Mean time to ulcer healing in the overall group was 160 days (SD 134); no statistically significant difference was found between the different offloading groups. A multivariate analysis was performed for time to ulcer healing, in which variables were included with a *p* < 0.15 (i.e., primary/recurrent ulcer, wound stage, PAD, location ulcer, number of ulcers, coronary artery disease, and type offloading). After correction, the following variables remained significant in correlation with time to ulcer healing: primary/recurrent ulcer (*p* = 0.014; hazard ratio [HR] = 0.670; 95% confidence interval [CI] = 0.487–0.923), wound stage (*p* = 0.004; HR = 0.623; 95%CI = 0.450–0.863), number of ulcers (*p* = 0.148; HR = 0.836; 95%CI = 0.655–1.006), and coronary artery disease (*p* = 0.067; HR = 0.696; 95%CI = 0.472–1.025).

Mean ulcer-free survival days in the overall cohort was 152 days. Patients treated with orthopaedic shoes had a mean 186 ulcer-free survival days, which was significantly more compared to patients treated with other offloading strategies (*p* = 0.034).

### 3.3. Treatment Outcome—Uncomplicated Ulcers

Outcomes of people with uncomplicated ulcers (i.e., Texas A1 or A2) are shown in Table 4. Diabetic foot ulcer healing after 12 weeks follow-up was 56% (n = 58); no statistically significant differences were found between the offloading device groups (*p* = 0.321). Diabetic foot ulcer healing after 20 weeks follow-up was 71% (n = 73); no statistically significant differences were found between the offloading device groups (*p* = 0.282). Diabetic foot ulcer healing after 1-year follow-up was 82% (n = 84); no statistically significant differences were found between the offloading device groups (*p* = 0.098).

**Figure 2:**
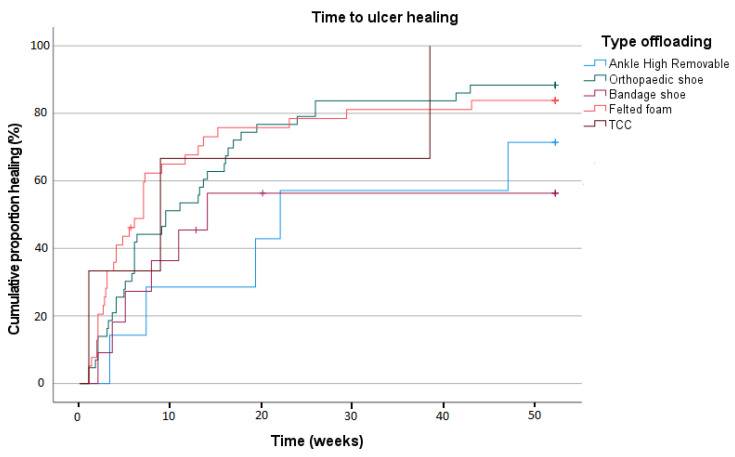
Time to ulcer healing in uncomplicated ulcers.

A Kaplan–Meier curve depicting time to healing is shown in Figure 2. Mean time to ulcer healing was 122.2 days (SD 12.8); no statistically significant differences were found between the offloading device groups (*p* = 0.287).

### 3.4. Treatment Outcomes—Complicated Ulcers

Outcomes of people with complicated ulcers (i.e., Texas A3 or Texas B, C, and D) are shown in Table 5. Diabetic foot ulcer healing after 12 weeks follow-up was 25% (n = 33); no statistically significant differences were found between the offloading device groups (*p* = 0.206). Diabetic foot ulcer healing after 20 weeks follow-up was 35% (n = 46); no statistically significant differences were found between the offloading device groups (*p* = 0.335). Diabetic foot ulcer healing after the 1-year follow-up was 65% (n = 86); no statistically significant differences were found between the offloading device groups (*p* = 0.117).

**Figure 3:**
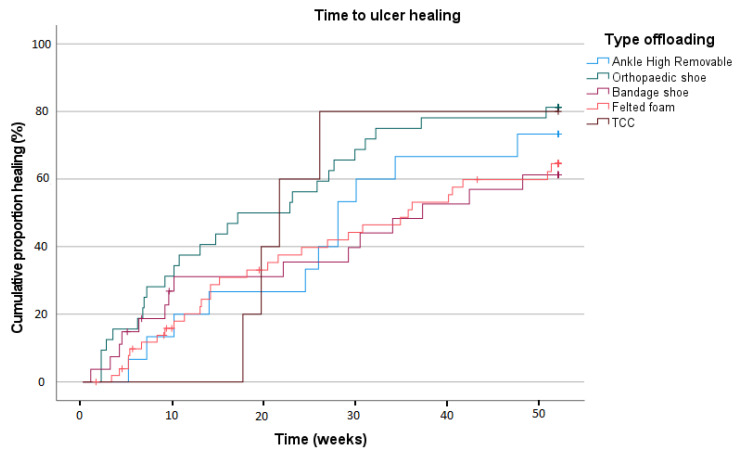
Time to ulcer healing in complicated ulcers.

A Kaplan–Meier curve depicting time to healing is shown in Figure 3. Mean time to ulcer healing was 207.5 days (SD 11.7); no statistically significant differences were found between the offloading device groups (*p* = 0.218).

## 4. Discussion

This study provides insights into daily clinical practice regarding the offloading devices used and the effectiveness of these devices in patients with a plantar diabetic foot ulcer in a multidisciplinary diabetic foot centre. Overall, we found 1-year healing rates of 77% for the total population, 82% for uncomplicated ulcers, and 65% for complicated ulcers. We will describe these three groups separately in this section, to better discuss the findings.

### 4.1. Total Population

Our patient population was consistent with previous characteristics from previous observational studies [9,16]. The majority of our patients had type 2 diabetes, and the vast majority had neuropathy, which is also the case in previous studies [9,16,39]. In addition, the patients in our cohort were on average 69 years old with multiple comorbidities such as ESRD, cerebrovascular disease, cardiovascular disease, and peripheral vascular disease. Almost a quarter (24%) of our patients had multiple ulcers on the feet and 18% had a deep ulcer (wound grade 3). All these factors reflect the daily practice in a diabetic foot reference centre of this complex group of patients with multiple factors that play a role in daily life and that also influence the treatment and healing of their diabetic foot ulcer.

Regarding the offloading methods used in our total population, knee-high non-removable (TCC) and ankle-high removable were used less frequently than other modalities. Previous observational studies also show that TCC is hardly used in practice (2–23%) [17,18,19,20,21]. This is consistent with our total population in which 3% received offloading by means of TCC. Patients in our cohort who received a lighter or less burdensome form of offloading, such as orthopaedic shoes, bandage shoes, or felted foam, were patients who were more vulnerable. These patients were significantly older (*p* < 0.001), more often had a history of cerebrovascular accident (*p* = 0.09), and more often had multiple ulcers (*p* = 0.002). Our findings seem to suggest that these factors were taken into account in the choice of offloading in daily practice, by choosing less burdensome offloading devices. Furthermore, patients with poorly controlled diabetes and type 1 diabetes more often received TCC or ankle-high removable offloading. This can be explained by the fact that these patients may be considered to have a higher risk of ulcers not healing and may be more likely to be non-adherent, or at least be assessed as such, causing the healthcare provider to opt for forced adherence by means of TCC. In the choice for offloading in the total population, we therefore seem to see that multiple ulcer-related factors and also patient-related factors were taken into account when determining offloading, resulting in a more personalized choice.

Regarding the healing outcomes in this retrospective cohort, we found a 77% 1-year healing rate in the overall group, which is comparable to healing rates in specialized tertiary hospitals in Europe [40,41]. However, there was a significant difference in the healing rate between the different offloading methods used, with patients treated with a bandage shoe having a lower 1-year healing rate than other offloading methods (*p* = 0.003). As described earlier, patients treated with a bandage shoe are vulnerable patients. Despite the complexity of the total population, it looks like a personalized choice of treatment pays off, as there are comparable results with previous observational studies in this area.

### 4.2. Uncomplicated Ulcers

With regard to offloading of uncomplicated ulcers, there is clear advice from the international guideline to use TCC as the first-choice offloading method [12]. In our cohort, however, only 3% of patients were treated with TCC and lighter forms of offloading were chosen more often. There are several possible explanations for the deviation from the first choice of the international guideline.

We know from previous studies that administering TCC as an offloading method can also have negative consequences on the quality of life and physical fitness of patients [18]. Another explanation could be that casting a patient with a TCC is a time-consuming procedure for the casting technicians. In this case, other offloading methods, such as felted foam, are often easier to apply in daily practice with many patients who are seen in the outpatient clinic.

Regarding the healing rates in the uncomplicated ulcers, we had an overall healing rate of 56% at 12 weeks follow-up. This is in line with a previous RCT in our centre using BTCC and other removable offloading methods with healing rates between 58% and 70% [33]. The ulcer healing rates at 12 weeks are comparable to those previously found for removable knee-high prefabricated walkers (52–79%), and for other healing shoes (43–70%) [11,22,23,42,43]. Our overall healing rate at 12 weeks was lower compared to other studies using non-removable offloading, which have reported healing rates of 83–95% at 12 weeks [15]. However, we cannot compare our healing rates at 12 weeks in the TCC group with healing rates from other studies because the number of patients in this group was very small in our study. Regarding the time to ulcer healing, compared to previous studies, our cohort showed a longer time to ulcer healing [15]. This can be explained by the fact that the vast majority in our cohort are treated with removable offloading devices.

### 4.3. Complicated Ulcers

The international guideline advises to offload patients with complicated ulcers based on patient-specific/individual factors. In our cohort of complicated ulcers, the majority was treated with lighter forms of offloading (felted foam, orthopaedic shoe, bandage shoe). Here too, patient and ulcer characteristics most likely played a role in making this choice. As shown in previous observational studies, different modalities are used, but in these studies, no separate percentages are given of these modalities in complicated ulcers [15,16,17,21].

Regarding the healing rates, 25% was healed after 12 weeks, but without significant differences between the offloading groups. The healing rate after 1 year was 65%, without significant differences between the offloading groups, with the majority of patients in the TCC group having a healed ulcer (80%). Both the chosen offloading methods in our cohort of complicated ulcers and the healing rate cannot be compared with other studies, as there is—to the best of our knowledge—no description of the specific healing results in complicated ulcers in any other study. The international guideline emphasizes that well-designed studies are urgently needed on offloading of ulcers other than the uncomplicated neuropathic plantar forefoot or midfoot ulcers to be able to better interpret our results [12]. It is essential to do more research into this because this patient category represents the majority of the daily practice in which we act.

### 4.4. Limitations

This study has some limitations. In our cohort, we did not include the analysis of other individual treatments such as adequate vascular surgical interventions in the results. The successful or unsuccessful treatment of peripheral artery disease plays an important role in ischemic wound and ulcer-related outcomes. Furthermore, a previous study has established the relevance of clinician judgment regarding the chosen treatment option and suggested that many uncontrolled variables (such as perceived adherence, patient preference, and patient mobility) may influence treatment choice and thus potential healing outcomes [44]. This results in a limitation of the ability of the previous referred study to determine the most effective offloading method. The same applies to the current study; these variables were not taken into account. In future studies, patient motivation should also be taken into account. Also, device-related adverse events were not documented and, therefore, it was not possible to evaluate their effect on the offloading strategies and the healing of diabetic foot ulcers. Furthermore, foot deformities or Charcot foot were not described in most of the patients’ status. This could influence the choice of offloading but unfortunately this was not possible in our cohort.

Another limitation is that this was a single-centre retrospective cohort study. Unfortunately, due to the retrospective aspect of this study, the choice of specific offloading devices could not be determined because the practitioner does not justify these choices in the patient files. Furthermore, another limitation in our study is the difference in size groups of the different offloading devices, which may also lead to a sampling bias. In addition, the relatively small number of patients per offloading device makes it difficult to draw a robust conclusion. Another limitation of this study is that in our clinic there is no step-by-step plan regarding offloading methods in which the first recommended and second recommended are given in case of a complicated diabetic foot ulcer. However, given the lack of well-founded evidence for the correct treatment of complicated ulcers, it is also not possible to make a comprehensive step-by-step plan for this selection of patients. The development of such a protocol is a great opportunity for future research.

## 5. Conclusions

Removable ankle-high offloading devices, orthopaedic shoes, bandage shoes, and felted foam are used most frequently in clinical practice. This seems to be the result of various physician- and patient-related factors such as logistical reasons, patient factors, and severity of complicated ulcers. Diabetic foot ulcer healing after 12 weeks, 20 weeks, and 1-year follow-up were consistent with previous observational studies. Well-designed studies are urgently needed with regard to the offloading of ulcers in real-life clinical practice to be able to better interpret our results.

## Figures and Tables

**Table 1:** Baseline characteristics and comorbidities of all patients.

		All Patients	TCC	Ankle-High Removable	Orthopaedic Shoe	Bandage Shoe	Felted Foam	*p*-Value
Patients		235	3% (n = 8)	9% (n = 22)	32% (n = 75)	16% (n = 38)	39% (n = 92)	
Gender	*Male*	66% (n = 156)	75% (n = 6)	86% (n = 19)	64% (n = 48)	63% (n = 24)	64% (n = 60)	0.311
	*Female*	34% (n = 79)	25% (n = 2)	14% (n = 3)	36% (n = 27)	37% (n = 14)	36% (n = 32)	
Age (years), mean (SD)		69.4 (12.4)	57 (11.0)	59.6 (11.9)	71.4 (11.0)	73.7 (11.2)	69.3 (12.5)	<0.001
Diabetes type	*Type 1*	5% (n = 13)	13% (n = 1)	23% (n = 5)	4% (n = 3)	0% (n = 0)	4% (n = 4)	0.003
	*Type 2*	95% (n = 222)	87% (n = 7)	77% (n = 17)	96% (n = 72)	100% (n = 38)	96% (n = 88)	
Years of diabetes		14.9 (10.4)	12 (5.7)	14.7 (11.6)	16.0 (12.2)	11.5 (6.9)	15.6 (9.6)	0.287
HbA1c		61.9 (17.6)	72.8 (20.2)	70.8 (21.4)	56.6 (13.4)	60.9 (19.8)	64.5 (17.9)	0.006
Hypertension	*Yes*	70% (n = 165)	75% (n = 6)	68% (n = 15)	67% (n = 50)	68% (n = 26)	74% (n = 68)	0.871
	*No*	30% (n = 70)	25% (n = 2)	32% (n = 7)	33% (n = 25)	32% (n = 12)	26% (n = 24)	
PAD	*Yes*	34% (n = 80)	0% (n = 0)	23% (n = 5)	23% (n = 17)	42% (n = 16)	46% (n = 42)	0.003
	*No*	66% (n = 155)	100% (n = 8)	77% (n = 17)	77% (n = 58)	58% (n = 22)	54% (n = 50)	
Cerebrovascular disease	*Yes*	16% (n = 37)	0% (n = 0)	9% (n = 2)	12% (n = 9)	29% (n = 11)	16% (n = 15)	0.090
	No	84% (n = 198)	100% (n = 8)	91% (n = 20)	88% (n = 66)	71% (n = 27)	84% (n = 77)	
Cardiovascular disease	*Yes*	29% (n = 69)	13% (n = 1)	18% (n = 4)	24% (n = 18)	34% (n = 13)	36% (n = 33)	0.217
	*No*	71% (n = 166)	87% (n = 7)	82% (n = 18)	76% (n = 57)	66% (n = 25)	64% (n = 59)	
ESRD	*Yes*	8% (n = 19)	0% (n = 0)	0% (n = 0)	8% (n = 6)	11% (n = 4)	10% (n = 9)	0.509
	*No*	92% (n = 216)	100% (n = 8)	100% (n = 22)	92% (n = 69)	89% (n = 34)	90% (n = 83)	
Neuropathy	*Yes*	86% (n = 203)	87% (n = 7)	82% (n = 18)	95% (n = 71)	82% (n = 31)	83% (n = 76)	0.157
	*No*	14% (n = 32)	13% (n = 1)	18% (n = 4)	5% (n = 4)	18% (n = 7)	17% (n = 16)	

Note: Values are % (n) or mean (SD). *p*-value compares the five types of offloading.

**Table 2:** Ulcer characteristics of all patients.

		All Patients	TCC	Ankle-High Removable	Orthopaedic Shoe	Bandage Shoe	Felted Foam	*p*-Value
Patients		235	3% (n = 8)	9% (n = 22)	32% (n = 75)	16% (n = 38)	39% (n = 92)	
Ulcer	*First ever*	59% (n = 138)	38% (n = 3)	54.% (n = 12)	53% (n = 40)	61% (n = 23)	66% (n = 61)	0.380
	*Recurrent*	41% (n = 97)	62% (n = 5)	46% (n = 10)	47% (n = 35)	39% (n = 15)	34% (n = 31)	
Number of ulcers	*1*	76% (n = 179)	63% (n = 5)	96% (n = 21)	88% (n = 66)	74% (n = 28)	64% (n = 59)	0.002
	*2*	13% (n = 30)	25% (n = 2)	4% (n = 1)	8% (n = 6)	3% (n = 1)	22% (n = 20)	
	*3*	6% (n = 15)	12% (n = 1)	0% (n = 0)	3% (n = 2)	10% (n = 4)	9% (n = 8)	
	*>3*	5% (n = 11)	0% (n = 0)	0% (n = 0)	1% (n = 1)	13% (n = 5)	5% (n = 5)	
Wound stage	*A*	45% (n = 105)	38% (n = 3)	36% (n = 8)	59% (n = 44)	29% (n = 11)	41% (n = 38)	0.001
	*B*	21% (n = 50)	62% (n = 5)	41% (n = 9)	19% (n = 14)	29% (n = 11)	12% (n = 11)	
	*C*	18% (n = 43)	0% (n = 0)	9% (n = 2)	9% (n = 7)	21% (n = 8)	29% (n = 27)	
	*D*	16% (n = 37)	0% (n = 0)	14% (n = 3)	13% (n = 10)	21% (n = 8)	17% (n = 16)	
Wound grade	*1*	52% (n = 123)	87% (n = 7)	32% (n = 7)	64% (n = 48)	37% (n = 14)	51% (n = 47)	0.019
	*2*	30% (n = 70)	13% (n = 1)	41% (n = 9)	19% (n = 14)	37% (n = 14)	35% (n = 32)	
	*3*	18% (n = 42)	0% (n = 0)	27% (n = 6)	17% (n = 13)	26% (n = 10)	14% (n = 13)	
Wound location	*Dig 1/Hallux*	43% (n = 100)	25% (n = 2)	59% (n = 13)	47% (n = 35)	40% (n = 15)	38% (n = 35)	0.281
	*Dig 2*	8% (n = 20)	0% (n = 0)	14% (n = 3)	9% (n = 7)	5% (n = 2)	9% (n = 8)	
	*Dig 3*	11% (n = 25)	12% (n = 1)	9% (n = 2)	11% (n = 8)	16% (n = 6)	9% (n = 8)	
	*Dig 4*	4% (n = 9)	12% (n = 1)	0% (n = 0)	7% (n = 5)	0% (n = 0)	3% (n = 3)	
	*Dig 5*	14% (n = 33)	25% (n = 2)	9% (n = 2)	15% (n = 11)	16% (n = 6)	13% (n = 12)	
	*Forefoot*	12% (n = 30)	25% (n = 2)	9% (n = 2)	9% (n = 7)	18% (n = 7)	13% (n = 12)	
	*Midfoot/Hindfoot*	8% (n = 18)	0% (n = 0)	0% (n = 0)	2% (n = 2)	5% (n = 2)	15% (n = 14)	

Note: Values are % (n). Ulcer stage and grade according to University of Texas Ulcer Classification [35].

**Table 3:** Treatment outcomes of all patients.

			All Patients	TCC	Ankle-High Removable	Orthopaedic Shoe	Bandage Shoe	Felted Foam	*p*-Value
Patients			235	3% (n = 8)	9% (n = 22)	32% (n = 75)	16% (n = 38)	39% (n = 92)	
Ulcer healing	12 weeks	*Yes*	33% (n = 78)	12% (n = 1)	18% (n = 4)	37% (n = 28)	29% (n = 11)	37% (n = 34)	0.255
		*No*	67% (n = 157)	88% (n = 7)	82% (n = 18)	63% (n = 47)	71% (n = 27)	63% (n = 58)	
	20 weeks	*Yes*	52% (n = 121)	25% (n = 2)	27% (n = 6)	59% (n = 44)	47% (n = 18)	55% (n = 51)	0.013
		*No*	48% (n = 114)	75% (n = 6)	73% (n = 16)	41% (n = 31)	53% (n = 20)	45% (n = 41)	
	1 year	*Yes*	77% (n = 180)	88% (n = 7)	73% (n = 16)	84% (n = 63)	66% (n = 25)	75% (n = 69)	0.003
		*No*	23% (n = 55)	12% (n = 1)	27% (n = 6)	16% (n = 12)	34% (n = 13)	25% (n = 23)	
Time to ulcer healing (days), mean (SD)			160.0 (134.2)	161.5 (112.7)	211.2 (126.0)	133.6 (121.7)	186.0 (145.4)	158.4 (139.9)	0.105
Ulcer recurrence		Yes	25% (n = 56)	37% (n = 3)	32% (n = 7)	31% (n = 23)	18% (n = 7)	21% (n = 19)	0.070
		No	75% (n = 171)	63% (n = 5)	68% (n = 15)	69% (n = 52)	82% (n = 31)	79% (n = 73)	
Ulcer-free survival days, median (IQR)			151.8 (134.7–169.0)	118.3 (23.9–212.6)	121 (72.4–170.1)	186 (157.4–215.4)	111 (65.7–155.9)	151 (122.0–179.6)	0.034

Note: Values are % (n), median (IQR) or mean ± SD.

**Table 4:** Treatment outcomes in uncomplicated ulcers.

			All Patients	TCC	Ankle-High Removable	Orthopaedic Shoe	Bandage Shoe	Felted Foam	*p*-Value
Patients			103	3% (n = 3)	7% (n = 7)	42% (n = 43)	11% (n = 11)	37% (n = 39)	
Time to ulcer healing (days), mean (SD)			122.2 (12.8)	112.3 (79.5)	202.5 (51.5)	111.7 (17.2)	188.1 (48.3)	104.9 (20.7)	0.287
Ulcer healing	12 weeks	*Yes*	56% (n = 58)	67% (n = 2)	29% (n = 2)	54% (n = 23)	45% (n = 5)	67% (n = 26)	0.321
		*No*	44% (n = 45)	33% (n = 1)	71% (n = 5)	46% (n = 20)	55% (n = 6)	33% (n = 13)	
	20 weeks	*Yes*	71% (n = 73)	67% (n = 2)	43% (n = 3)	77% (n = 33)	55% (n = 6)	74% (n = 29)	0.282
		*No*	29% (n = 30)	33% (n = 1)	57% (n = 4)	23% (n = 10)	45% (n = 5)	26% (n = 10)	
	1 year	*Yes*	82% (n = 84)	100% (n = 3)	71% (n = 5)	88% (n = 38)	55% (n = 6)	82% (n = 32)	0.098
		*No*	18% (n = 19)	0% (n = 0)	29% (n = 2)	12% (n = 5)	45% (n = 5)	18% (n = 7)	

Note: Values are % (n) or mean ± SD. Uncomplicated ulcers are ulcers that are not infected/ischemic, and are superficial (Texas A1 and A2).

**Table 5:** Treatment outcomes in complicated ulcers.

			All Patients	TCC	Ankle-High Removable	Orthopaedic Shoe	Bandage Shoe	Felted Foam	*p*-Value
Patients			132	4% (n = 5)	11% (n = 15)	24% (n = 32)	21% (n = 27)	40% (n = 53)	
Time to ulcer healing (days), mean (SD)			207.5 (11.7)	191.0 (39.7)	215.3 (30.0)	163.1 (22.1)	227.2 (27.6)	227.9 (18.8)	0.218
Ulcer healing	12 weeks	*Yes*	25% (n = 33)	0% (n = 0)	20% (n = 3)	38% (n = 12)	30% (n = 8)	19% (n = 10)	0.206
		*No*	75% (n = 99)	100% (n = 5)	80% (n = 12)	62% (n = 20)	70% (n = 19)	81% (n = 43)	
	20 weeks	*Yes*	35% (n = 46)	40% (n = 2)	27% (n = 4)	50% (n = 16)	30% (n = 8)	30% (n = 16)	0.335
		*No*	65% (n = 86)	60% (n = 3)	73% (n = 11)	50% (n = 16)	70% (n = 19)	70% (n = 37)	
	1 year	*Yes*	65% (n = 86)	80% (n = 4)	73% (n = 11)	81% (n = 26)	56% (n = 15)	57% (n = 30)	0.117
		*No*	35% (n = 46)	20% (n = 1)	27% (n = 4)	19% (n = 6)	44% (n = 12)	43% (n = 23)	

Note: Values are % (n) or mean ± SD. Complicated ulcers are ulcers that are deep ulcers or infected/ischemic (Texas A3, B, C, and D).

## Data Availability

The data presented in this study are available on request from the corresponding author.

## Appendix A

1 TCC.

2 MABAL shoe.

3 FOS.

4 Pulman bandage shoe.

5: Orthopaedic shoe.

## Appendix B

	**Healing 20 Weeks**			
	Univariate	*p*-value	Multivariate	*p*-value
	odds ratio (CI-95)		odds ratio (CI-95)	
Age	1.008 (0.987–1.029)	0.451		
Diabetes type	0.827 (0.269–2.538)	0.740		
Years of diabetes	0.999 (0.973–1.026)	0.956		
HbA1c	0.990 (0.975–1.006)	0.231		
Hypertension	1.037 (0.593–1.814	0.899		
PAD	*0.227 (0.126–0.409)*	*<0.001*		
Cerebrovascular	0.703 (0.346–1.426)	0.329		
Cardiovascular	*0.304 (0.167–0.553)*	*<0.001*	*0.438 (0.224–0.857)*	*0.016*
ESRD	0.688 (0.266–1.777)	0.440		
Neuropathy	1.191 (0.564–2.513)	0.647		
Ulcer (first/recurrent)	0.803 (0.477–1.351)	0.409		
Number of ulcers	*0.499 (0.338–0.738)*	*<0.001*	*0.593 (0.381–0.922)*	*0.020*
Wound stage	*0.491 (0.378–637)*	*<0.001*	*0.641 (0.474–0.867)*	*0.004*
Wound grade	*0.448 (0.311–0.646)*	*<0.001*	*0.596 (0.392–0.905)*	*0.015*
Wound location	*0.902 (0.801–1.015)*	*0.088*		
	**Healing 1 Year**			
	Univariate	*p*-value	Multivariate	*p*-value
	odds ratio (CI-95)		odds ratio (CI-95)	
Age	0.992 (0.969–1.015)	0.477		
Diabetes type	0.852 (0.253–2.871)	0.797		
Years of diabetes	0.991 (0.962–1.020)	0.542		
HbA1c	1.003 (0.985–1.021)	0.778		
Hypertension	0.964 (0.515–1.804)	0.908		
PAD	*0.406 (0.225–0.732)*	*0.003*		
Cerebrovascular	*0.432 (0.209–0.892)*	*0.023*	*0.436 (0.203–0.936)*	*0.033*
Cardiovascular	*0.420 (0.230–0.786)*	*0.005*	*0.494 (0.259–0.940)*	*0.032*
ESRD	0.814 (0.296–2.241)	0.691		
Neuropathy	0.854 (0.362–2.011)	0.718		
Ulcer (first/recurrent)	1.286 (0.714–2.315)	0.402		
Number of ulcers	*0.601 (0.431–0.838)*	*0.003*	*0.690 (0.483–0.985)*	*0.041*
Wound stage	*0.687 (0.534–0.885)*	*0.004*		
Wound grade	*0.622 (0.431–0.899)*	*0.011*	*0.669 (0.454–0.984)*	*0.041*
Wound location	*0.891 (0.783–1.014)*	*0.080*		
